# The dataset of proteins specifically interacted with activated TICAM-1

**DOI:** 10.1016/j.dib.2016.06.030

**Published:** 2016-06-28

**Authors:** Kenji Funami, Misako Matsumoto, Hiroyuki Oshiumi, Chikashi Obuse, Tsukasa Seya

**Affiliations:** a Department of Microbiology and Immunology, Graduate School of Medicine, Hokkaido University, Sapporo 060-8638, Japan; b Division of Molecular Life Science, Graduate School of Life Science, Hokkaido University, Sapporo 001-0021, Japan

**Keywords:** TLR3, TICAM-1 (TRIF), 14-3-3, Signalosome, Proteome analysis

## Abstract

The presented data are related with our paper entitled “14-3-3-zeta participates in TLR3-mediated TICAM-1 signal-platform formation” (Funami et al., 2016) [1]. These data show the proteins which specifically bind to the activated (oligomerized) TICAM-1. Fifty-three proteins were identified as specifically interacted with oligomerized TICAM-1. Mutant TICAM-1 cannot form the active oligomer, so the proteins interacted with mutant TICAM-1 are dispensable for TICAM-1-signaling. Among 53 proteins, 14-3-3-zeta specifically interacts with oligomerized TICAM-1 to corroborate TICAM-1 signalosome.

**Specifications Table**

**Table:** 

Subject area	*Biology*
More specific subject area	*Innate immunity*
Type of data	*Table, figure*
How data was acquired	*Mass spectrometry analysis*
Data format	*Analyzed data in excel file*
Experimental factors	*TICAM-1 binding proteins were co-immunoprecipitated from HEK293 cells transfected with full-length TICAM-1 and non-specific binding was subtracted.*
Experimental features	*Immunoprecipitated samples were separated by SDS-PAGE and subjected to LC/MS/MS analysis.*
Data source location	*Sapporo, Hokkaido, Japan*
Data accessibility	*All data are accessible in this article.*

**Value of the data**

• This data shows the components of TICAM-1 signalosome, which are important for the regulation of TICAM-1 functions.
• This data first distinguishes a ‘functional binding’ to the activated TICAM-1 from ‘off-target binding’ to a non-functional mutant, TICAM-1-N+TIR-P434H.
• This data shows that several types of 14-3-3 proteins are identified as TICAM-1-binding proteins. In addition to TICAM-1-signalosome formation we identified, 14-3-3 proteins may have other functions in the field of innate immunity.

## 1 Data

**Fig. 1:**
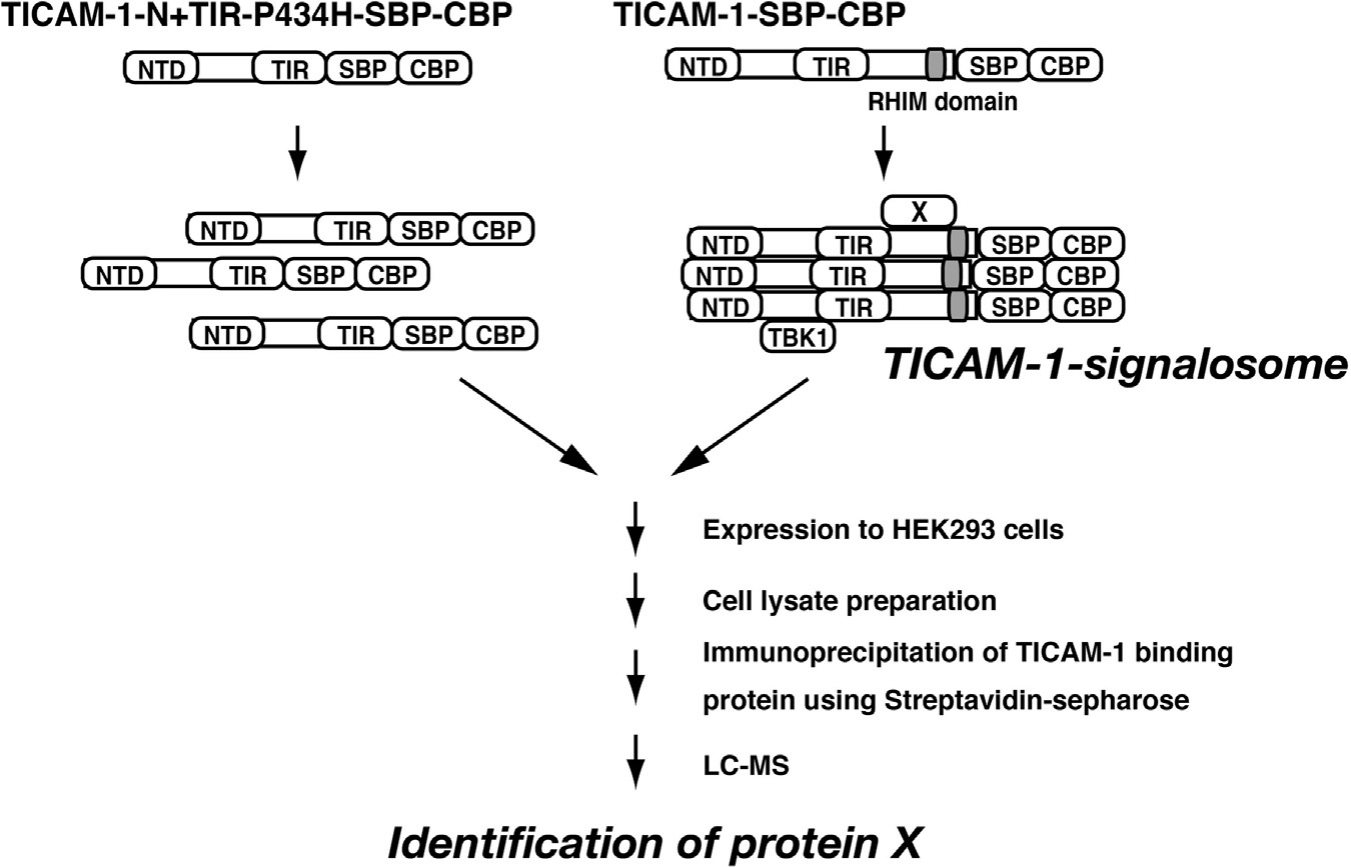
Fetching TICAM-1-binding protein using LC-MS-MS analysis.

We show the strategy for identification of TICAM-1-signalosome component in Fig. 1. By mass spectrometry, we identify the proteins interacted with functional TICAM-1 and non-functional TICAM-1 mutant, and represent the list of the proteins in Table 1.

## 2 Experimental design, materials and methods

Full-length TICAM-1 and TICAM-1-N+TIR-P434H were expressed in HEK293 cells and TICAM-1-binding proteins were immunoprecipitated by Streptavidin Sepharose (GE healthcare) [1]. Eluted samples were separated in a 10% SDS-polyacrylamide gel for liquid chromatography coupled to tandem mass-spectrometry (LC/MS/MS). The raw data files obtained from the LC/MS/MS were analyzed as described previously, with minor modifications [2], [3]. Our scheme was depicted in Fig. 1.

The TICAM-1 construct having Tags (streptavidin-binding peptide (SBP) and calmodulin binding peptides (CBP)) and that lacking the RHIM domain with a mutated TIR domain were provided as reported previously [4]. The latter fails to oligomerize to activate RIP1 kinase in transfected cells. TICAM-1-signalosome-binding proteins were obtained by subtraction using proteome analyses (LC/MS/MS). NTD, N-terminal domain; TIR, toll-IL-1β-homology domain.

## Footnotes

Transparency document Transparency data associated with this article can be found in the online version at doi:10.1016/j.dib.2016.06.030.
