# Supplementary material for: The dataset of proteins specifically interacted with activated TICAM-1
Source: Data Brief. 2016 Jun 28;8:697–9. doi: 10.1016/j.dib.2016.06.030 (PMC4949732; doi:10.1016/j.dib.2016.06.030)
Supplement: Supplementary file 2 — Supplementary material [file mmc2.doc]

**Re: DIB-D-16-00315**

**Conflict of interest**

The authors declare no financial of commercial conflict of interest.
